# A scheduling perspective on modular educational systems in Europe

**DOI:** 10.1016/j.heliyon.2024.e39694

**Published:** 2024-10-29

**Authors:** Rubén Ruiz-Torrubiano, Sebastian Knopp, Andreas Krystallidis, Lukas Matthias Wolf

**Affiliations:** a IMC Krems University of Applied Sciences, Piaristengasse 1, Krems, 3500, Austria; b Untis GmbH, Belvederegasse 11, Stockerau, 2000, Austria; c Planimize, Gardanne, 13120, France

**Keywords:** Modular education, Scheduling, Timetabling

## Abstract

In modular educational systems, students are allowed to choose a part of their curriculum themselves. The rationale behind letting students choose their courses themselves is to enhance self-responsibility, improve student motivation, and allow for focus on specific areas of interest. A central instrument for bringing these systems to fruition is the timetable. However, scheduling the timetable in such systems can be an extremely challenging and time-consuming task. In this study, we present a framework for classifying modular educational systems in Europe that reflects different degrees of freedom regarding student choices and explore the consequences from the perspective of scheduling a timetable that satisfies all requirements from the organizational and pedagogical perspectives. For this purpose, we conducted interviews in Austria, Germany, Finland, Switzerland, the Netherlands, and Luxembourg, and applied the framework to these educational systems, finding that among them the Finnish system shows the highest degree of modularity. After analyzing the consequences of modularity from the scheduling perspective, we assess the necessity for automated scheduling methods, which are central to realizing the potential and many benefits of modular education. The framework developed in this paper can be used by educational systems to assess their degree of modularity and consider the right approach to timetabling based on it.

## 1 Introduction

Curriculum design is one of the central activities of stakeholders involved in educational systems to steer and implement strategic goals that education should fulfill in a given country or region. One of the central goals is to meet the current needs, demands and expectations of society, especially students, parents and teachers. Depending on the stakeholders and the output of this activity, different levels of curriculum design can be defined [1]: international/comparative (*supra*), system/nation/state (*macro*), school or institution (*meso*), classroom (*micro*) and individual level (*nano*). The international or supra level is usually associated with comparative studies (e.g., PISA) and reflects on aims and quality of education in general. The macro level is focused on the particular implementation of those goals in a given societal context. This is normally achieved in the form of national syllabi or concrete objectives. The meso level is concerned with the implementation of the curriculum in the context of a particular school. For instance, a school might decide to offer additional electives or make decisions on how parts of the curriculum are delivered. Finally, the micro and nano levels are mostly concerned with the classroom implementation of the curriculum and how it is handled by teachers and students.

As a matter of fact, these levels are interrelated and cannot be considered in isolation. In particular, one important aspect is how to enhance student engagement (i.e., the nano level) already at the macro level. In practice, this means laying the regulatory groundwork for improving student involvement and participation at the lower levels. One of the most effective approaches in this context is the introduction of electives in the curriculum, which is often referred to as modular course design. The origins of modularization in education can be traced back to Harvard University in the 19th century [2], where an elective system was introduced to increase the choices available for students and increase the flexibility of the curriculum. This development was explicitly encouraged in Britain in the 1960s and 1970s to increase participation rates and enhance teacher and student mobility. Recently, modular education has been shown to significantly decrease school dropout rates [3] and improve student achievement [4].

Evidently, the implementation of modular education following the macro level has to traverse the meso (school or institution) level, which can be thought of as the ‘operational’ level. At this level, one of the most challenging operational questions is how to schedule these modules (i.e. courses) in a timetable, possibly alongside other, non-modular components. It has been shown that how courses are scheduled can affect the academic performance of students [5]. From the organizational perspective, the timetable can be considered a central resource plan that coordinates how educational resources (teachers, rooms, laboratories, etc) are used. Constraints arising from a variety of sources, including teacher and room availability, student timetable quality and pedagogical considerations can make timetabling a very challenging task.

### 1.1 Previous work

The term *module* is defined in [3] as an autonomous component that bundles a set of learning outcomes. In a modular educational system, modules are assigned a set of competencies or skills which can be chosen by students with a varying degree of autonomy. The introduction and implementation of modular education in vocational education in Germany is described in [6]. The authors argue that modularization is an effective approach for skill and qualifications development. In Germany, the main goals of modularization are found to be in this case flexibilization and individualization. A comparison of the modularization approaches of Germany, Austria and Switzerland is given in [7]. The effect of modular education on student achievement and other outcomes has been the subject of several studies. In [3], a statistically significant reduction in school dropout is observed in vocational education in the region of Flanders, Belgium. The motives that drive students to choose some modules over others was investigated in [8]. The authors find that intrinsic motivations are more important for young students than extrinsic ones.

Several authors have pointed to the need for automation support in the development and implementation of modular curricula. In the context of educational timetabling, the authors of [9] review the organizational and computational challenges of the process of building timetables. An interactive course-timetabling system in a modular curriculum setting is discussed in [10]. The authors describe the complexity associated with building a timetable in such a setting and their practical implications. In [11] the authors present an ontology-based model for curriculum management, which is especially tailored for modular curricula. Ontologies are a way of structuring knowledge that capture elements and their relationships in a principled way. The authors extract this ontology from curriculum documents and use it as a tool for curriculum development. In [12], an approach based on artificial intelligence (AI) for competence-based curriculum design is investigated and apply their method in the area of engineering education. A process for developing a curriculum management system and its benefits is described in [13].

In contrast to most studies in this area, which usually focus on higher education, our work specifically addresses the needs of secondary education institutions in general, not limited to vocational education only as in several other studies [3], [6], [7]. In general, there is a lack of studies on both modular educational systems and the need for automated administration and scheduling solutions.

### 1.2 Aims of this study

In this study, we aim to answer the following research question: *“How do different educational systems in Europe implement modular education at the secondary level and how does this relate to timetable scheduling from an organizational and pedagogical perspective?”*. We note that the focus of our work is placed on secondary rather than tertiary education. To answer the research question, a novel theoretical framework is developed to assess the degree of modularity in those systems qualitatively and quantitatively. We focus on the educational systems of Austria, Germany, Finland, the Netherlands, Switzerland and Luxembourg and perform semi-structured interviews with experts and practitioners to gather insights into how modularity influences scheduling and what are the most important problems in practice. This framework is the first step in a larger effort to propose efficient computational methods for solving the scheduling problem in modular educational systems in practice. To the best of our knowledge, this is the first study that investigates modular education from a scheduling perspective.

The main contribution of this article is a new qualitative framework for classifying and analyzing educational systems at the secondary education level based on their degree of modularity. Based on this framework, we will derive insights regarding their practical implementation, focusing on the problem of how to build the corresponding timetables. Finally, we will apply our framework to several European educational systems and compare them using a novel category system.

The rest of this article is organized as follows. In Section 2 we outline the methodology used for this study and introduce central definitions used throughout the paper. Section 3 presents the results of the interviews and the proposed framework. A detailed discussion of our findings is provided in Section 4 and we conclude in Section 5 with a summary and future work.

## 2 Methodology

### 2.1 Research approach

We perform a qualitative content analysis [14] based on Mayring's approach [15]. The main difference between this approach and standard content analysis is that quantitative analysis (e.g., frequency analysis) is not used as a means for evaluating the resulting categories. Instead, a systematic, theory-guided approach is used for text analysis that takes the context into account. Therefore, the focus of the analysis is on the qualities, the processes and the meanings rather than on experimentally measurable entities. Furthermore, Mayring's approach is theory-guided, which allows the integration of previous knowledge and theoretical frameworks into the analytic process. This method is well-suited to answer our research question because we expect to find different views on qualities in the educational systems investigated. Additionally, qualitative content analysis is well suited when the data sources are heterogeneous, like in the present case where the interviews (our primary source of information) are completed with regulatory texts from different regions and other materials.

**Figure 1:**
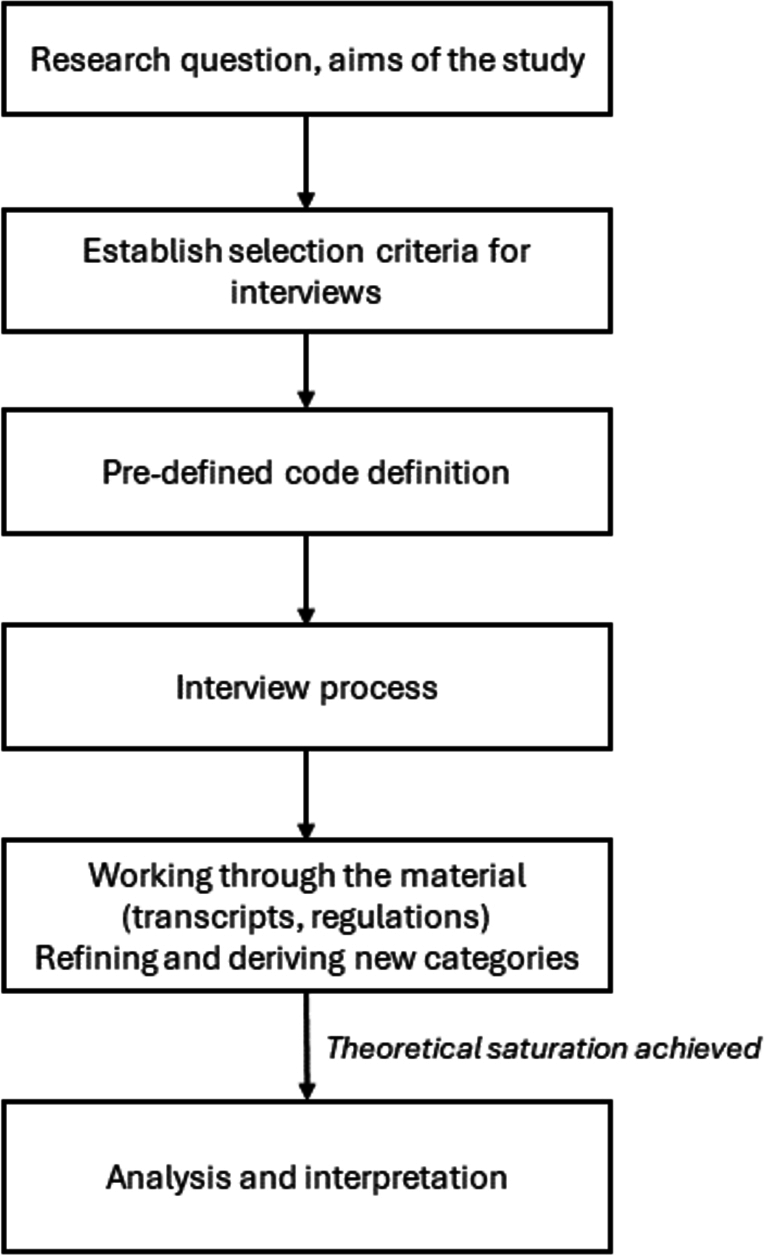
Schematic representation of the research approach used in this study.

Our research approach is depicted in Fig. 1. We start by setting the research question and the aims of the study. Then, the selection (resp. exclusion) criteria for the interviews were defined. These criteria are detailed in Subsection 2.2. Before the interviews were conducted, we defined a set of pre-defined codes, as explained in Subsection 2.3. After that, we conducted semi-structured interviews with 13 experts in total, as we describe in the next subsection. The interviews were then transcripted, and the process of refined coding and category definition started. During this process, we identified the categories explained in Section 3. Finally, we analyzed the results and drew the conclusions of the study.

Our work can be framed as an instance of case study research. The goal of case study research is to investigate phenomena within their context [16]. Case study as a research strategy is particularly well suited when the subject of the investigation includes organizational or managerial processes [17], as in the current context. Our focus is on the organizational processes dedicated to scheduling in the context of the particular modular educational system under consideration. Therefore, we conducted semi-structured interviews with 13 experts with direct experience in scheduling timetables in modular educational systems. The interviews were performed from January until July 2023. Additionally, interview notes and internal documents were also incorporated into the analysis. After conducting the interviews, an inductive approach was used to develop and refine the categories. Coding was performed collaboratively by iteratively achieving mutual consent regarding the results of the analysis in what is known as communicative validation [15].

### 2.2 Interview partners and regions

In Table 1 we provide a summary of all the interview partners involved, including a short description of their expertise and the country/region they work in. The criteria used for including experts in our study were the following:

• Working in the region considered for more than 5 years.
• Having knowledge of the regulations involved in their educational system.
• Having experience with the problem of scheduling timetables.
• Optionally, having experience with specific timetabling tools.

**Table 1:** Summary of interview partners.

ID	Country (region)	Description	Experience
E1	Austria	High school timetabler	More than 5 years
	(Lower Austria)	(*Gymnasium*)	
E2	Germany	Timetabling-software expert	More than 25 years
	(Baden-Wurttemberg)		
E3	Germany	High school timetabler and	More than 25 years
	(Baden-Wurttemberg)	timetabling-software expert	
E4	Germany	High school timetabler and	More than 25 years
	(Bavaria)	timetabling-software expert	
E5	Germany	Timetabling-software expert	More than 15 years
	(Lower Saxony)		
E6	Germany	High school timetabler	More than 25 years
	(Rheinland-Palatinate)		
E7	Germany	Modular education expert	More than 25 years
	(Rheinland-Palatinate)	at local Ministry	
E8	Netherlands	High school timetabler	More than 25 years
E9	Luxembourg	High school timetabler	More than 5 years
		(*European school*)	
E10	Luxembourg	High school timetabler	More than 10 years
		(*European school*)	
E11	Switzerland	Timetabling-software expert	More than 25 years
	(German-speaking)		
E12	Switzerland	Timetabling-software expert	More than 10 years
	(French-speaking)		
E13	Finland	Project manager	More than 10 years
		(educational software), high	
		school timetabler	

The interviews were limited to 45 minutes each. First, the interview partner was asked to give an overview on the applicable regulations in their educational system. Then, questions were asked about the practical challenges involved, specifically when building the timetables. To conclude, some time was reserved for open-ended remarks and personal reflection. As can be seen from Table 1, seven of the experts are timetablers and therefore with relevant practical expertise in the organizational processes of the school. Additionally, six interview partners are experts in timetabling software with extensive knowledge of their respective modular education systems. Finally, one of the interview partners works at the local Ministry of Education and contributes with extensive knowledge of the educational system. Note that in total eight out of the total of thirteen experts come from a German-speaking country, and several German regions are represented separately. The reason is that the authors already have extensive experience with the educational systems in these regions. Additionally, the federal nature of the German educational system makes it necessary to investigate regions separately, as differences in curricula and school types can be significant. The other countries investigated (Austria, Finland, the Netherlands, Luxembourg and Switzerland) were chosen as the availability of materials and interview partners for those countries could be managed as a part of the study and the authors have more domain knowledge about these educational systems.

### 2.3 Coding of the interviews

Once conducted, the interviews were transcripted and annotated. Since the authors have experience with different educational systems, this *a priori* knowledge was incorporated into the coding process as a set of pre-defined codes. These codes included terms like “election”, “module”, “scheduling” and “choices”. Once the interviews were encoded using these terms, the codes were refined to take recurrent themes like “election systems”, “class level overlaps”, “number of choices”, etc, into account. The refined codes were obtained inductively by finding commonalities and recurrent themes between the contents of the different interviews. For instance, it was a common theme to describe what the system of electives looked like in terms of number of electives or student choices made.

After going through several iterations of this method, consensus was reached on the final categories including a categorization of each educational system into this set of categories. In Section 3, we present the results of this categorization in detail. In Section 4, we analyze and discuss these results.

### 2.4 Basic definitions

In this section, we define some basic terms that will be used later on to classify the modular education systems that we study in this paper.

**Class**: A group of students that attend all regular lessons together. By regular lessons, we mean those that are mandatory for all students (i.e., they cannot be elected).
**Class level/grade**: The yearly part of a class, e.g., the fourth grade, the fifth grade, etc. Typically, individual classes belong to a given class level (i.e., the classes 4a, 4b, etc.). In what follows we will refer to class levels meaning groups of classes with the same yearly part.
**Lesson**: A lesson is an event where a group of students belonging to one or several classes are taught in a given subject. Usually, the total regular weekly duration of a lesson is split into sessions that can have different lengths. For instance, a math lesson can have a regular duration of 3 hours per week and be split into one two-hour and one one-hour session. Sessions are normally constrained by standard times defined in a *time grid*. For instance, a school might define that each day lessons start at 8.00 am and each session has a duration of 50 minutes. In this case, instead of talking about hours, we will normally talk about *time slots* or *periods* (both terms are interchangeable).
**Courses/Electable lessons**: Lessons are called electable if they are not mandatory for a student, i.e., they can be selected eventually subject to some restrictions (e.g., pre-conditions like previous knowledge/attended courses). In this paper, we will use the term course to mean an electable lesson.
**Student election/choice**: We will refer to the action of a student selecting a course/module as an election or choice (these terms can be used interchangeably).
**Term**: This is a subdivision of a school year into disjoint intervals. Most typically, a term can represent a semester (e.g., fall/spring semesters), but other, shorter terms are also possible.

## 3 Results

We now describe the category system resulting from encoding the case-study interviews presented in Section 2. First, we describe the category system, which is composed of a total of eight categories. Then, we apply these categories to the individual educational systems that were investigated in the conducted interviews by evaluating each category in the specific context of each system. In Section 4, a visual comparison between the investigated educational systems regarding their degree of modularity is provided along with a detailed discussion of the results.

### 3.1 Categories

After conducting the qualitative content analysis and coding process described in Section 2, the following categories were identified:

**Proportion of electable periods (PEP)**: Each modular educational system investigated in this study shows varying degrees regarding the proportion of electable lessons. Given a class level, we define the proportion of electable periods in that class level as the total number of periods of courses that can be elected by students in that class level divided by the total number of periods of all lessons (electable and mandatory). So for example, if there are 5 lessons in class level 8, and 2 of the 5 lessons are courses, and all lessons have a duration of 2 periods, the PEP of class level 8 would be (2⋅2)/(5⋅2)=0.4, or 40%. We take the maximum of all class levels as the PEP of a given school form. E.g., if in one class level every course can be freely elected, we consider this as 100%, even if in lower class levels nothing can be chosen. By using the maximum, we provide an accurate picture of the potential of a given modular system to provide electable lesson times.
**Election systems (ES)**: In general, there are different ways of offering electable lessons to students. After conducting the interviews, we distinguish between three different module selection systems:
  **Class-based**: Each student is assigned only to lessons that are planned for the class the student belongs to.
  **Profile-based**: Each student can choose one out of a list of pre-defined *profiles*. A profile determines a set of courses that all students who have chosen the same profile take together. For example, it might be possible in a given school to choose between *natural sciences* and *humanities*. The profile *natural sciences* could include the courses mathematics, physics and chemistry and *humanities* could include Greek, Latin and philosophy. All the other lessons are taught class-based (i.e. are mandatory).
  **Individual**: Each student can choose among a pool of courses individually with a given level of freedom. Consequently, each course can be composed of a different group of students that might be unique for that course. Note that in individual election systems, it might also be the case that some proportion of the lessons are taken class-based (i.e., core subjects like mathematics and history).
  As will be shown later, it is also possible to have profile-based systems where students can choose additional subjects outside of their profile (e.g. in the Netherlands). We consider these systems to be halfway between a profile-based and a pure individual election system.
**Number of student elections (SE)**: During their stay at school, students in modular systems will need to make several elections for courses taught at a given class level (e.g. by filling out some forms). For example, if students are asked to select courses once in each of the last three years, we define the number of student elections to be three.
**Proportion of module-oriented class levels (ML)**: This is the proportion of class levels offering either profile-based or individual course elections. For example, if there are 5 class levels and 3 of them offer electable lessons, this proportion would be 3/5=0.6 or 60%.
**Number of terms per year (T)**: The number of different periods of time characterized by different regular timetables. In systems where there is a fall and a spring semester, the number of terms per year would be 2. In other systems (e.g. Finland) there might be an arbitrary number of terms higher than 2.
**Scheduling workflow (SW)**: We identified two different scheduling workflows from the experts for creating timetables in modular systems. Either the timetable is created first and students choose courses based on the known timetable or students select courses based only on their preferences without knowing the timetable in advance. In this last case, the timetable is created considering all student elections. We call the first workflow the *timetable-first* and the second the *election-first* workflow.
**Proportion of courses with class level overlap (CLO)**: We say that a course shows *class level overlap* if students from different class levels are allowed to attend the course (i.e., the course is electable for students in different class levels). For instance, a philosophy course might be offered as electable for all students in the seventh and eighth grades, which means that potentially students from both levels can be taught that course simultaneously.
**School course collaborations (SC)**: In some systems, rare or unusual subjects requiring teachers with special qualifications might be offered collectively by two or more schools to reduce costs and optimize attendance. In this case, guest students from other schools are allowed to attend a course given in a specific host school.

Table 2 presents a general overview of our category system. In addition to the name of the category, the abbreviation, and a short description, the last column specifies which range of values each category is allowed to take. For proportions, in general, we divide the range of possible values into three intervals *low*, *medium* and *high* that correspond each to one-third of the [0,100] interval. Cardinal values are positive integers. All the other categories take values in a definite set (e.g., “Yes/No”). In the following, we review the secondary modular educational systems of Austria, Finland, Germany (for selected federal states), Luxembourg, the Netherlands and Switzerland. For a list of abbreviations used other than the categories (which are specified in Table 2), we refer the reader to Table 3.

**Table 2:** Summary of the resulting categories.

Category Name	Abbr.	Description	Values
Prop. electable periods	PEP	How many periods are	Low, medium,
		electable?	high
Election systems	ES	Which framework is used for	Class, profile,
		electable courses?	individual
No. student elections	SE	How often have students to	Low, medium,
		select courses?	high
Module-oriented class levels	ML	How many class levels	Low-medium,
		contain modular courses?	high
No. terms/year	T	How many different terms	Cardinal
		take place in a school year?	
Scheduling workflow	SW	How is the timetable	Timetable-first,
		scheduled?	Election-first
Prop. courses with class	CLO	How many courses are mixed	Low, medium
overlap		among class levels?	high
School collaborations	SC	Are courses offered among	Yes/No
		different schools?

**Table 3:** Other abbreviations used in this paper.

Abbreviation	Meaning (original)	Meaning (translated)
AGY	Allgemeinbildendes Gymnasium	General high school
		(Baden-Wurttemberg/Germany)
BGY	Berufliches Gymnasium	Vocational high school
		(Baden-Wurttemberg/Germany)
BOS	Berufsoberschule	Vocational-oriented
		high school (Bavaria/Germany)
ESC	Enseignement secondaire	Classical secondary education
	classique	(Luxembourg)
ESG	Enseignement secondaire	General secondary education
	général	(Luxembourg)
FOS	Fachoberschule	Subject-oriented high school
		(Bavaria/Germany)
HAVO	hoger algemeen voortgezet	General secondary education
	onderwijs	(Netherlands)
MOST	Modulare Oberstufe	Modular upper cycle
		(Austria)
NOVI	Neue Oberstufe mit verstärkter	Modular upper cycle with
	Individualisierung	strengthened individualization
		(Austria)
OS	Oberstufe	Upper cycle
		(Austria and Germany)
SOST	Semestrierte Oberstufe	Upper cycle with semesters
		(Austria)
VMBO	Voorbereidend middelbaar	Preparatory secondary vocational
	beroepsonderwijs	school (Netherlands)
VWO	Voorbereidend wetenschappelijk	Pre-university education
	onderwijs	(Netherlands)
US	Unterstufe	Lower cycle
		(Austria and Germany)

### 3.2 Austria

In Austria, secondary education is distributed among different school types [18]. The type of high school known as *Gymnasium* is the most relevant from the point of view of modular education. In general, class levels are grouped into two cycles of four years each, the lower cycle (or *Unterstufe*, US) from the first until the fourth grade and the upper cycle (or *Oberstufe*, OS) from the fifth until the eighth grade. From the sixth until the eighth grade there is a minimum of four and a maximum of ten hours per week of electives that can be offered by each school autonomously. Modular education is still under development in Austria. One of the earliest forms of modularization in secondary education was the MOST (modular upper cycle), which has been tried individually at some schools since the late 2000s. This school form was unified in what was called NOVI (new upper cycle with strengthened individualization), which is being tested at the time of this writing in about 30 schools. This model is planned to evolve into the SOST (upper cycle in semester-based form), which changes the modules' assessment from a yearly to a semester basis [19].

In the NOVI, there are up to eleven elective modules that can be chosen without any pre-conditions. These eleven courses have a duration of one semester and are typically taught for two hours per week and distributed evenly among the four semesters of the last class levels in the OS (seventh and eighth grade). That means that, in a typical week, students attend about 30 hours of non-electives and 4-6 hours in modules. To facilitate scheduling, all non-electives are planned in the morning, and the modules are taught in the afternoon.

*Category classification.*  According to our category system, the proportion of electable periods can be considered to be on the lower end of the scale. Using the typical scenario outlined before, this would amount to about one-third of the total number of periods per week to be in modules. The module election system is individual: there are no restrictions for students on which modules they are allowed to select. The number of choices that a student has to make lies within the lower end of the scale (students only choose their subjects once and this remains until the end of the upper cycle). In this system, only the last two class levels are module-oriented, i.e., from the total of eight levels that would result in a total proportion of 25%. In a typical year, there are two semesters (fall/spring). Regarding scheduling workflow, first, the timetable is made, and after that, the students can select their subjects based on the already existing schedule. Class level overlaps are usual (students from the seventh and the eighth grade can be present) and there are no school collaborations.

### 3.3 Finland

Finland has a long tradition of modular education at the upper secondary level [20], comprising the last class levels where students are generally between 16 and 18 years old. High schools therefore normally encompass three years although some students have to take a fourth. All students participate in the elective course system. Courses are not necessarily bound to class levels and therefore most courses can be elected by students in any of them. We see around 75% elective courses in course election data, the others being mandatory.

*Category classification.*  The choice system is individual, except for general class hours with a class teacher in one subject. The number of choices is as follows: students in the first two class levels choose between 30-40 courses, while students in the third and last year can select between 20-24 courses. There are between 5-6 terms per year and courses can take place in any of them, but students also can take a certain exam so the corresponding course has to take place in the corresponding term too. Students first make their course choices, and afterwards the timetable is created (election-first workflow). Usually, the assignment of teachers to courses is not known at the time the student make their choices. School collaborations are usual, especially in the context of a given municipality. High schools in the same municipality share courses that students from other schools can attend. This results in a very high complexity when scheduling courses, since times for shared courses need to be taken into account.

### 3.4 Germany

In Germany, the responsibility for the school system lies with the 16 federal states. Consequently, significant differences between them exist. As in Austria, secondary education is distributed among different school types. Regarding modular education, school types that lead to the general qualification for university entrance (*Abitur*) are the most relevant. These are of the types *Gymnasium*, *Oberschule*, or *Gesamtschule*. In particular in the two or three highest class levels (upper cycle) electives can be chosen by students with a high degree of freedom.

In the following subsections, we present the situations in specific federal states. We conducted five interviews with seven experts for course timetabling. These interviews cover schools in 10 of the 16 federal states of Germany: Two experts for Baden-Württemberg, one expert for Bavaria, three experts for Rhineland-Palatinate, and one expert covering seven states (Brandenburg, Bremen, Lower Saxony, Mecklenburg-Vorpommern, Saxony, Saxony-Anhalt, Thuringia) at the same time.

#### 3.4.1 Baden-Württemberg

In Baden-Württemberg, there are two different school types in secondary education. One is the *Allgemeinbildendes Gymnasium* which has an elective system that allows many individual electives for students. The other is the *Berufliches Gymnasium* where more periods per week (about 12 to 14) take place within a class context and a profile-based election system is used. In both school types, a course-based system is used in the final two year levels. In Baden-Württemberg, there are either 12 or 13 year levels for each student including primary school. So, depending on this, the course-based system is used in year levels eleven and twelve, or twelve and thirteen.

##### Category classification.

As *Allgemeinbildende Gymnasien* (AGY) and *Berufliche Gymnasien* (BGY) are quite different, we classify them in separate categories. The proportion of electable periods is medium (BGY) or high (AGY). All periods take place in modules (AGY) or roughly half of the periods (BGY). The module election system is individual (AGY) or profile-based (BGY). The remaining category classification is identical for both school types. Students make their choices once, right before they enter the class levels of the upper cycle. So, the number of elections is low. In this system, only the last two class levels are module-oriented, i.e., from the total of eight (or nine) levels that would result in a total proportion of 25% (low). Timetables can be different in each term (half-year). An election-first scheduling workflow is used. Students first make their course choices, and after that, the timetable is created. The assignment of teachers to courses is not known at the time the student make their choices. Class-level overlaps can be planned for lessons that are chosen by only a few students. This happens rarely (low). For the same reason, some schools in this state collaborate to offer common courses.

#### 3.4.2 Bavaria

In the federal state of Bavaria, modular education is mainly present in two school types: the *Fachoberschule* (FOS) and the *Berufsoberschule* (BOS). The main difference lies in the practical orientation: while the FOS can be attended by all students with a middle school diploma, the BOS is intended for students with some professional experience that aims at deepening their training in that area [21]. Both types of schools lead to the general qualification for university entrance. The FOS comprises three class levels (11, 12 and 13), while the BOS encompasses only two (12 and 13). Students choose electives at the eleventh class level, having more choices in the BOS than the FOS. In general, besides general subjects like math, history and ethics, students can choose one out of seven different profiles, each with their respective profile subjects (e.g. environmental sciences, health sciences, technical subjects, etc.). Furthermore, and based on the chosen profile, students can choose up to two additional elective courses that aim at deepening the knowledge of the student in a particular direction (for instance, diverse language courses, biotechnology, arts and music, etc.). In total, around 20 hours per week are dedicated to general subjects, 10 for profile subjects and 4 hours for freely electable modules.

##### Category classification.

The proportion of electable periods can be considered medium (about 50% of the time is spent in either profile or electable courses). Therefore, the module election system is a mixture of profile-based and individual systems. Students typically choose their profile and electives once (in the eleventh class level in the FOS or during their practical training in the BOS), and these elections remain constant until their graduation. All class levels except the eleventh (FOS) are modular, and there is generally no distinction between semesters. The scheduling workflow is election-first: students choose their profiles and electives first, and then the timetable is made to fulfill all the student elections. Class-level overlaps are possible, and in general, there are no school collaborations.

#### 3.4.3 Rhineland-Palatinate

Modular education in Rhineland-Palatinate is mainly applied in the last three years of the *Gymnasium*, which can be either the 10th, 11th and 12th grades in 8-year schools (G8) or the 11th, 12th and 13th grade in 9-year schools (G9). Across all class levels, students take between 32 and 35 hours per week of courses, which are divided into basic courses (which are taught for 3 hours per week) and advanced level courses (which are taught for 4 or 5 hours per week). In general, a student will choose 3 advanced courses, the rest being basic courses on subjects not considered central regarding the profile of the student or the school itself. In practice, resources are bundled together in some cases by defining advanced courses as basic courses with additional hours where more advanced material is taught.

The course election process is normally coordinated by a single person, whereas another person is in charge of scheduling the courses in the timetable. Each year, students are asked to elect courses until March, and then groups of courses that can be scheduled together are clustered. Later on, the timetable is made according to those clusters. If possible without creating any disadvantage for other students, elected courses can be changed until the next autumn.

##### Category classification.

The *Gymnasium* upper cycle in Rhineland-Palatinate can be considered as a medium-level modular system based on individual student elections. The core of this system is given by the election of the advanced courses. Since those courses are taught for either 4 or 5 hours per week, that results in between 12 and 20 hours per week spent on advanced courses, which roughly amounts to half the weekly lesson load for a typical student. Only class levels in the upper cycle are modular, resulting in about 1/3 of modular class levels in the Gymnasium, and also here there is no distinction between semesters. The scheduling process here is, as typical in Germany, also election-first. Class level overlaps are usual, and some schools may collaborate with others by bundling educational resources.

#### 3.4.4 Brandenburg, Bremen, Lower Saxony, Mecklenburg-Vorpommern, Saxony, Saxony-Anhalt, Thuringia

Given that one of our interview partners is an expert on the educational systems of these federal states, and since these are quite similar, we proceed to report on all of them in this section. In general, we can distinguish between profile-based (e.g. in Bremen) and individual-election systems (e.g. Lower Saxony). In the former case, a high portion of the courses students attend are fixed by their profiles, whereas in the latter, students choose their courses individually. Depending on the specific federal state, the Gymnasium is divided into 9 class levels (G9), mostly in Bremen and Lower Saxony, or 8 levels in the rest (G8). Similarly as in Rhineland-Palatinate, only typically the last three levels are modular. While in the 11th grade a typical student spends about half of their time in modular courses, the 12th and the 13th grade (where appropriate) is completely modular, so students are allowed to elect all of their courses. Typically, students will choose in the 11th class their courses, and once again in the 12th.

##### Category classification.

In this case, we can regard these federal states as having highly modular educational systems, since the average amount of hours spent on modular courses by the average student is higher than 50%. There is a mixture of profile-based and individual election systems, although individual systems are more common. Like in Rhineland-Palatinate, about a third of all class levels in the Gymnasium can be regarded as modular. The scheduling process is election-first, with frequent class-level overlaps and the possibility of school collaborations (mostly when these schools are located in the same city).

### 3.5 Luxembourg

The secondary school system in Luxembourg starts with 12-year-old students and is divided into the following variations: classical secondary education (ESC), general secondary education (ESG), European education, International English curriculum, British education and German-Luxembourgish education. The high school education cycle in all cases always comprises seven class levels [22]. Since both interviewees E9 and E10 work at European schools, we will focus on this variant in the following discussion. In the European system, the 7-year cycle (S1-S7) is itself subdivided into three sub-cycles S1-S3, S4-S5 and S6-S7. In general, the degree of modularity increases with each sub-cycle. As part of the individual educational approach of each school (school autonomy), schools may introduce additional subjects or classes tailored to the particular approach of that school.

*Category classification.*  The proportion of electable periods starts in the first sub-cycle S1-S3 with approximately 30% of periods in modular courses (including language courses) and increases to 50% in S4-S5 until about 90% in the last two years. The election system used is based on individual choices (no profiles are given). Students make course selections a total of three times, one per sub-cycle. All seven class levels of the secondary education system can be considered modular, therefore the proportion of modular class levels is the highest. There is no special distinction between different terms in the course of a regular school year. The scheduling workflow used is an election-first type of workflow where students select their courses first, and then the timetable is scheduled according to the student's choices. In general, students from different sections and class levels can be mixed into individual courses, like languages and natural sciences. School collaborations are not usual, since some schools are already divided into different sites and this would overly complicate the scheduling process.

### 3.6 Netherlands

The secondary education system in the Netherlands is divided into pre-vocational (VMBO), pre-university (VWO) and senior general secondary education (HAVO) [23]. The VMBO is profile-oriented. Once students choose a profile (an occupational sector), a predefined set of courses is automatically assigned. In general, students need to attend between 3 and 4 mandatory courses that make up a total of about 15 hours per week for a typical student. In the HAVO and the VWO, students attend a pre-defined curriculum for the first three years. In the upper cycle, this common part of the curriculum gets reduced to core subjects like Dutch, English and social studies. Students then choose one among four different profiles (technology, health, economics and culture). However, students are allowed to choose up to two additional subjects related to the profile, and one additional elective, which might be unrelated to the profile. Regarding the timetable, schools try to assign students to courses so that the students stay in the same group most of the time and avoid mixing students from different class levels (which is usual in other countries like Germany). On the one side, this facilitates scheduling the different courses to a high degree, and on the other side helps students keep their social bonds during the time they spend in secondary school.

*Category classification.*  The degree of modularity of the different secondary education branches is slightly different for VMBO, VWO and HAVO, but in general, one can consider the system to be medium modular. In all types of secondary education, there are core subjects and optional subjects, which are always pre-determined by the choice of a profile. Therefore, secondary education in the Netherlands can be regarded as profile-based. The number of choices that the pupils make is either only one (in fully profile-based variants like VMBO) or up to two (like in the VWO). All class levels contain electives to some degree, and in general, there is no distinction between fall and spring semesters. The schedule is made in an election-first manner, and if possible no students from different class levels are mixed in individual courses. School collaborations are not usual, since secondary schools in the Netherlands tend to be already quite large.

### 3.7 Switzerland

For Switzerland, we conducted two interviews: one for the French-speaking cantons, and one for the German-speaking cantons. We present the results in separate sections, as there are considerable differences between both approaches.

#### 3.7.1 German-speaking cantons

In the German-speaking cantons of Switzerland, modular education is mainly present at community schools (*Gemeindeschulen*). Most of the schools in these cantons are of this type, overall the number of such schools is around 1000. Although Gymnasiums also exist in these cantons, they almost don't offer any modular education, therefore we don't consider them further in this paper. In the community schools, and all schools that implement the compulsory part of the educational system, modular education is concerned basically with the last class level (that is the third level of the lower secondary cycle). However, there is no general obligation for schools to offer elective courses [24]. When they do, choices are made by students in spring before the timetabling takes place in summer. Students choose specific courses without specifying alternatives. In general, there is no collaboration between schools to offer shared courses. It is considered to be very important that idle periods must be avoided in the timetables of students.

##### Category classification.

The proportion of electable periods is highly variable, but in general, the system can be considered to be of low modularity. The module election system is individual. In general, students choose only once (in the last year of their compulsory education). The proportion of module-oriented class levels is therefore in general limited to one class level out of nine. Timetables can be different in each term (half-year). An election-first scheduling workflow is used. Since in general only one class level is involved, there is no class level overlap in the elective courses. As mentioned above, in general, there are no school collaborations for shared courses.

#### 3.7.2 French-speaking cantons

There are in total eight French-speaking cantons in Switzerland. In general, secondary education is divided into a lower and an upper cycle. The lower cycle is common for all of Switzerland and lets students choose among three predefined profiles. However, the upper cycle (which involves students of ages between 15 and 19 years) can be tailored to specific cantons reflecting the diversity of approaches. In our interview, we focused on the Geneva, Fribourg, and Jura cantons as they stand out for their high degree of modularization. Before entering the upper cycle, students are asked to select a basic set of courses, including two languages and a main optional subject (students can specify alternatives in case the elected course is full or not available for some other reason). Students might also request to attend a course at another school, which in general is possible if they stay in the same canton. The mandatory subjects (French, geography, history and mathematics) account for about 30% of the total lesson time for a typical student. Students are asked to choose courses again in their third year (in the upper cycle), but the scope of these electives is reduced compared to the first election. Scheduling is done by first finding a good time assignment for all the courses and then assigning the students to courses.

##### Category classification.

We can consider the upper secondary education system in the above-mentioned cantons to be modular to a high degree (i.e. about 70% of the lesson time is spent in elective courses). Students choose subjects twice during the upper cycle, once at the very beginning and once again in the third year. While students are mostly free to choose their subjects, it results in practice into a large number of profiles (i.e. possible combinations of electives). However, our interview partner confirmed that from about 250 students, the number of resulting profiles can be as high as 200 or above. Therefore, for this study we consider this system to be individually oriented, rather than profile-oriented. The proportion of modular class levels is 100%, since both the lower and the upper cycle are designed in a modular way. There is no distinction between terms and the scheduling workflow is timetable-first (seemingly for historical reasons). Students from different class levels can be mixed in principle, and collaborations between schools are possible (e.g. a student attending courses in another school of the same canton).

## 4 Discussion

As can be seen in the interviews, the approaches to modular secondary education are very heterogeneous. However, some common aspects can be identified. For example, all of the aforementioned educational systems use modular education as a means of fostering individualization and flexibilization in students' curricula. On the one side, individual-oriented election systems (like Germany or Finland) give more weight to students to follow their interests and deepen their understanding of core subjects. These electives might be relevant in their career after leaving secondary education, but professional usefulness alone does not provide the main rationale for the system. On the other side, profile-based systems (like in the Netherlands) are more usually focused on vocational education, and profiles are crafted in such a way that students learn to know which professions might be relevant in their professional lives. In between, there are many different approaches to modular education as represented for instance in the case of Austria. In Germany, there are plans to harmonize the modular education system in the Gymnasium upper cycle to focus more on the advanced courses (as interview partner E7 mentioned) rather than basic courses, stressing the focus of the system as an enabler to deepen students' understanding of core subjects. How successful these plans turn out to be depends on future developments. As can also be seen in the case of Switzerland, countries with a highly federal structure tend to fragment their educational systems, making harmonization difficult in the long run.

**Figure 2:**
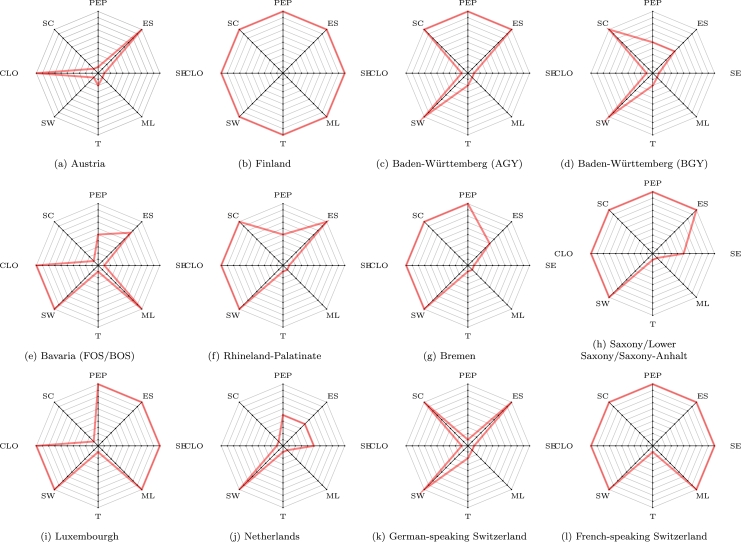
Visual comparison using radar charts from the educational systems studied in this paper.

Fig. 2 shows the results of the classification of all educational systems investigated in this work graphically. For this purpose, we use radar charts, which depict the different categories as axes where higher values (i.e. modularity-increasing) are represented as points with higher distance from the center. For example, the higher the modularity regarding the PEP (proportion of electable periods, see Table 2) parameter, the higher the distance to the center of each chart. The resulting shape (delineated using red lines) represents geometrically the degree of modularity of each educational system taking all the parameters into account. Ideally, the larger this shape, the more modular that particular educational system is according to our categories. Note that we use a minimum of one unit for each axis to improve readability. According to this representation, the most modular educational system is Finland (Fig. 2b), followed by French-speaking Switzerland in Fig. 2l (particularly, the Geneva canton), with Lower Saxony and related German federal states (Fig. 2h), Bremen (Fig. 2g) and Rhineland-Palatinate (Fig. 2f) thereafter. Finland scores the maximum scores in all categories investigated. In general, highly-modular systems have a high degree of electable periods in common (medium in case of Rhineland-Palatinate) and individual-oriented election systems, with frequent class-level overlaps. Interestingly, they also share an election-first approach to scheduling. Other systems like Austria (Fig. 2a) follow a more conservative approach with previously determined slots in the timetable for electives and a smaller proportion of electable courses. It can also be remarked that during our interview, expert E1 mentioned not believing that an election-first approach could be feasible, given the sheer amount of different possibilities to consider. However, such an approach has been practiced successfully since at least the 90s in other parts of Europe, notably in Germany. We therefore need to stress here the important role of automated timetabling tools in delivering support for complex modular educational systems. It is also important for experts to know what automated timetabling tools can do in this area.

### 4.1 Consequences for scheduling

The interviews show that there are various challenges when trying to create a schedule that satisfies all the needs of students, teachers and schools. The central problem shared by all modular educational systems studied is how to assign courses to students in such a way that students can attend all the courses they have elected. For instance, there might not be enough teachers for courses where too many students registered, or courses might need to be canceled when not enough students decide to enroll. In these cases, it will be necessary to relocate students to other courses. Additionally, course occupation needs to be balanced as well, e.g. if two advanced math courses are equivalent (i.e. parallel courses), it should be avoided that the number of students that attend each course is very different.

Another aspect that came up frequently in the interviews was the necessity to minimize the number of idle periods for students (and, secondarily, for teachers as well). In class-based systems, since students attend lessons together, the timetabler can easily ensure that core times are always assigned a lesson, resulting in no idle times for the entire class. However, this can be significantly more challenging in modular systems, since every student has a (potentially) different timetable. As a result, some timetablers (mainly in Germany) use a two-step method for building the schedule: First, they cluster courses in such a way that these courses can be scheduled simultaneously (no teacher or student clashes). Second, they maximize the number of students in every cluster, which means that when a cluster is scheduled in a given time slot, the number of students having idle times will be minimized. If these clusters can fill a set of core time slots, it can be guaranteed that as few idle periods as possible are produced. However, this task can be extremely challenging and most interview partners agreed that automated tools are needed. In some systems (e.g. the Netherlands), timetablers try to avoid class-level overlaps to make this problem easier to solve to some extent. Even then, it might be impossible to avoid idle times at all. How difficult these problems turn out to be is largely determined by the PEP (proportion of electable periods) and ES (election system) categories in our framework. In general, the higher the PEP, the more possibilities need to be considered when building the timetable. The more individualized an election system, the more difficult it is to consider all student choices.

Lastly, the room situation and teacher availability need to be considered as well. If too many courses are scheduled at the same time that need the same type of room (e.g. chemistry lab, projectors, etc), the timetable might be infeasible. Similarly, teachers working at more than one school need to be considered separately since they might not be available on certain days or times. Modular systems with school collaborations face this type of problem when building timetables (e.g. in Finland) more often.

In general, we conclude that the higher the modularity as holistically estimated by our framework, the more difficult building a timetable becomes. Not only does our framework capture this degree of modularity (which is linked to the difficulty of the timetable), but also allows to differentiate between educational systems in the individual categorical axis defined. Additionally, we can also spot similarities in how modularization is implemented across different systems. When building automated timetabling tools, this framework can be used to translate those commonalities and differences into mathematical language and use state-of-the-art computational techniques to support the scheduling process. Note that we do not argue here for a single timetabling system that can be used in all the regions investigated. Our goal was to introduce a language to use when building such tools in a unified way.

The question remains of how specifically build automated timetabling solutions that include support for modular educational systems. In the remaining of this section, we introduce different timetabling problems and specifically ask if the problems we identified in the interviews can be solved using these existing frameworks.

### 4.2 Existing timetabling frameworks

The problem of creating a timetable that satisfies a given set of requirements is not new and has been studied for more than 50 years [25]. Throughout the years the problem of educational timetabling has evolved into three subproblems that are often treated as independent: high school timetabling, course timetabling and examination timetabling [26].

In high school timetabling the focus lies in creating a weekly schedule for the various lessons of classes in a way that gives both students and teachers a compact schedule according to their availabilities while also paying attention to room assignments and other organizational constraints.

Course timetabling centers around the assignment of students to classes that are then scheduled in a timetable where each week of the term is scheduled individually. Again many different organizational constraints like assigning rooms and paying attention to the availabilities of teachers apply but the main focus lies in scheduling the lessons in a way such that every student can attend their desired courses. This makes it a good fit for creating schedules for universities.

Finally, in examination timetabling the goal is to assign exams to examination periods in a way that every student can take the exams they signed up for. This problem differs from the others since examinations are usually singular events without patterns that extend over several weeks or months. However, many of the constraints from other educational timetabling problems, like room requirements and distributing events, still apply.

From the interviews we have conducted, we can conclude that none of the three mentioned categories completely cover the requirements for the regions that we investigated. For instance, course timetabling focuses on the assignment of students to classes, usually not considering other aspects like compactness or pedagogical considerations like scheduling lessons in double periods. Both student assignments and pedagogical considerations are relevant in all educational systems studied. The problem definition seems to lie somewhere between high school timetabling and course timetabling. However, combining those two disciplines is no easy task since each on its own already is difficult enough to inspire decades of research that still results in non-optimal schedules. Nevertheless, we want to move the research in a direction that focuses on more modern school systems with modular course choices for students. To achieve this, this study represents the first step in a series of papers where we will propose new techniques for improving automatic scheduling software. We hope that developing such automatic timetabling software that supports the requirements outlined in this paper will make it easier for schools to transition to modular education which in turn will give students many more opportunities to follow their interests and prepare for their future careers through individualized choices.

### 4.3 Limitations

In general, theoretical saturation was achieved at the interviews, since no additional insights emerged from them, and the conceptual categories were explored in detail and are complete [27], [28]. For this investigation, we had access to interview partners covering a wide range of educational systems. However, the studied regions by far don't cover the whole of secondary education in Europe. We believe the selection presented in this paper to be representative of the main trends in modern modular education, but prominent examples (like the United Kingdom, France, and other parts of Western Europe) are missing. We plan to fill this gap in future work.

Another limitation of our work is that most timetable experts already work with timetabling tools, and therefore their insights could be biased towards that particular tool. During the interviews, we tried to keep the discussion independent of the particular tools used by the experts and focus on the organizational problems and the regulatory frameworks. In future work, a more diverse set of experts should also be included in the interview process.

## 5 Conclusion

In this paper, we presented a novel framework for evaluating the degree of modularity of different educational systems at the secondary level across eight different categories, which allows comparing them on a principled basis. These categories include the proportion of electable periods, the type of election systems and the number of student choices that need to be made, among others. After applying this category system to the educational systems of different European countries, we find that the Finnish system offers the greatest degree of modularity. Specifically, we investigate how this degree of modularity affects scheduling the timetable. Using this category system and the insights obtained from expert interviews, we argue that automated tools are needed to support the practical implementation of modular educational systems.

To the best of our knowledge, this is the first effort of this kind in the literature to date. This study lays the groundwork for analyzing the needs and requirements of timetabling in different regions when modular courses are present, paving the way for a detailed investigation into how to automate these requirements to build specially tailored tools that support realizing the promise of modular education in a way that improves its quality for all stakeholders involved, including students, teachers, school administration and policymakers.

As future work, automatic timetabling methods that comply with the requirements investigated in this paper using the proposed framework will be developed to support educational systems across Europe to provide and extend modular education at the secondary level. Additionally, the validity of our framework will be tested and evaluated by including more experts from other regions and educational systems than the ones already investigated in this study.

## CRediT authorship contribution statement

**Rubén Ruiz-Torrubiano:** Writing – review & editing, Writing – original draft, Visualization, Validation, Supervision, Project administration, Methodology, Investigation, Funding acquisition, Formal analysis, Conceptualization. **Sebastian Knopp:** Writing – review & editing, Writing – original draft, Methodology, Investigation, Conceptualization. **Andreas Krystallidis:** Writing – review & editing, Investigation. **Lukas Matthias Wolf:** Writing – review & editing, Writing – original draft, Methodology, Investigation, Conceptualization.

## Declaration of Competing Interest

The authors declare that they have no known competing financial interests or personal relationships that could have appeared to influence the work reported in this paper.
